# The management of allergic rhinitis by pharmacists in public services: a proposed PhaRmacISt-led Education Model (AR-PRISE)

**DOI:** 10.1186/s40545-022-00477-1

**Published:** 2022-11-08

**Authors:** Chii-Chii Chew, Chee-Tao Chang, Xin-Jie Lim, Wai-Yin Yong, Doris George, Pathma Letchumanan, Philip Rajan, Chee Ping Chong

**Affiliations:** 1 Discipline of Clinical Pharmacy, School of Pharmaceutical Sciences, Universiti Sains Malaysia, 11800 Minden, Penang Malaysia; 2 School of Pharmacy, Monash University Malaysia, Subang Jaya, Malaysia; 3 Clinical Research Centre, Hospital Raja Permaisuri Bainun, Ministry of Health, Ambulatory Care Centre (ACC), Level 4, Jalan Raja Ashman Shah, 30450 Ipoh, Perak Malaysia; 4 Department of Pharmacy, Hospital Raja Permaisuri Bainun, Ministry of Health, Ipoh, Perak Malaysia; 5 Department of Otorhinolaryngology, Hospital Raja Permaisuri Bainun, Ministry of Health, Ipoh, Perak Malaysia

**Keywords:** Allergic rhinitis, Public health service, Pharmacist, Patient education, Educational model

## Abstract

Allergic rhinitis has been identified as a major respiratory disease that places a significant burden on patients and the healthcare system. Nevertheless, the management of allergic rhinitis is challenging for both patients and practitioners. Pharmacists have been recognised as strategic in providing advice for allergic avoidance, disease information, and pharmacological care for allergic rhinitis management. This role has been underutilised in the public health service sector in Malaysia due to variation in practice, regulation, and health system structures when compared to the international guidelines. This article proposed a PhaRmacISt-led Education Model (AR-PRISE) that includes explicit patient education materials and an algorithm for structured counselling by pharmacists in the management of patients with allergic rhinitis.

## Introduction

Allergic rhinitis has been recognised as one of the major respiratory diseases, involving chronic inflammation of the nasal membrane and commonly manifests as nasal congestion, nasal itching, rhinorrhoea, and sneezing [1]. Globally, it affects 10–30% of adults and 40% of children, and the prevalence has been increasing in the last two decades [2, 3]. In Malaysia, the prevalence of 21–24% in adults and 18.8% in adolescents are reported [4, 5].

The burden attributed to allergic rhinitis management has been significant. Notably, the indirect costs of management, such as absenteeism and presenteeism from school or work, have been greater than the direct costs of treatment for this disease. The indirect cost is estimated to be 5.2 billion USD vs. 3.4 billion USD (direct cost) per year in the U.S. A similar trend of economic burden is seen in most countries (Table 1) [6]. The economic burden in Malaysia has not been comprehensively reported so far.

**Table 1:** Prevalence of allergic rhinitis in adults and its associated economic burden

Country	Prevalence	Direct cost	Indirect cost
United States	15–30% [3]	3.4 billion USD per year [6]	5.2 billion USD per year [6]
Europe	21% [2]	€159 to €554 per patient/year [6]	€2405 per untreated patient/year*[14]
Sweden	32% [15]	€ 210 per patient/year [6]	€751 per patient/year [6]
Spain	11.4% [2]	€ 554 per patient/year [6]	€1772 per person/year
France	13% [2]	€159 per patient/year [6]	€543 per patient/year [6]
Turkey	27.7% [16]	79 USD per patient/year	Not available
Korea	13.3% [17]	224 USD million/year [6]	49 USD million/year [6]
India	20–30% [18]	215 USD per patient/year [6]	460 USD per patient/year[6]
China	16.8–23.9% [19]	€ 195.6 patient/year [20]	€ 440.9 patient/year [20]
Malaysia	21–24%[4]	Not available	Estimation: 2195 USD per patient per year[21]

*Allergic diseases including allergic rhinitis, allergic asthma, atopic dermatitis, urticaria and contact allergies

The factors contributing to the burden of this disease can be examined through the lenses of patients and healthcare providers. In the patients’ perspectives, the study found that patients self-managed the disease based on their own experiences. They tend to seek information from a variety of sources, including family, friends, spouses, and healthcare providers [7]. Patients do not fully understand this disease, are dissatisfied with the treatment, and a cure is not available for the problems encountered [8]. They commonly regard allergic rhinitis as a minor issue, resulting in non-adherence to pharmacotherapy and self-medicating when symptoms flare [9, 10].

The healthcare providers routinely dispensed medication and provided recommendations, but very few follow-up opportunities were observed, and patients frequently continued to adopt this advice over the long term without evaluation [7]. The Allergic Rhinitis and its Impact on Asthma (ARIA) guidelines have been used by health care professionals internationally for allergic rhinitis management [1, 11]. A lack of knowledge of ARIA among pharmacists is observed as evidenced by insufficient counselling and treatment selection by pharmacists in allergic rhinitis management [12]. In a recent study, it was found that 40.4% of Malaysian pharmacists have inadequate awareness of the ARIA guidelines [13].

## Fundamental management strategy

The ARIA guidelines emphasise that the fundamental approach to allergic rhinitis management is firstly having patients understand the overall aspect of this disease, instead of focusing on the use of intranasal administration techniques [22]. This includes patients' having an adequate understanding of the disease nature and progression, pharmacotherapy, and non-pharmacological management (allergen avoidance). To get the patient to buy-in the concept of allergic rhinitis management, the fundamentals of the management should always be started with patient education, which has been reiterated by ARIA guidelines since 2008 [1].

Improvement in the understanding of allergic rhinitis would help patients change their expectations of medication treatment from expecting "a total cure of disease" to "disease control". Concise, unbiased, and accurate information could improve patients' understanding of their medication, its benefits and risks, and generate realistic expectations, all of which would promote adherence. Subsequently, the progression to complications of allergic rhinitis, such as chronic rhinosinusitis or worsening of underlying asthma control, which results in a higher burden to the health system, can also be avoided [7, 10, 23].

Nonetheless, patient education materials with regard to allergic rhinitis management have been unstandardized and varied in their styles and comprehensiveness. In the U.K., the patient leaflets had no publication date (47%), were at least 5 years (30%), contained inadequate information for management and therapy (42%), and contained inaccurate information (79%) [24]. Besides, a study in Australia shows that patients sought advice from various resources, including pharmacists, family and friends, the internet, and the media. Of concern, some of the information obtained is often inadequate, inconsistent, fragmented, and has not been reviewed by the subject matter experts [7]. In Malaysia, online information on allergies has not been updated periodically [25].

## A local standard of pharmaceutical care

The widely used ARIA pharmacy guidelines focus on managing allergic rhinitis by community pharmacists [22]. In western countries, most people with allergic rhinitis seek healthcare services from community pharmacists. The community pharmacy management algorithm begins with disease identification, then severity assessment, and, if needed, primary care practitioner referral. This structure might not be feasible in countries practising private and public services, such as the Malaysian health system. The lack of a dispensing separation policy in private primary healthcare limits community pharmacists' involvement in Malaysia [26]. Specific allergic rhinitis management guidelines in the local setting are lacking [27]. The ARIA pharmacy management recommends creating a local standard of care by addressing variances in pharmacy practice, infrastructure, personnel, and regulations in each country [11].

## Role of pharmacists

Studies have shown that pharmacists are impacting the care of chronic diseases by improving adherence to proper medication regimens, a key factor in the improvement of patient outcomes.

The pharmacists have been described as having a key role in providing allergic rhinitis management information beyond medication advice [22]. These include disease identification, allergen avoidance strategies, risk–benefit of treatment, promoting patient self-management, patient support, and follow-up care. They are perceived to have time to educate patients and the ability to improve health outcomes, especially in cases with suboptimal control [7, 22, 28]. "Pharmaceutical services" from pharmacists who have been trained in allergic rhinitis management improve patients' symptom control and quality of life. [12].

## AR-PRISE model

Recognising the importance of pharmaceutical care and the potential expansion of the roles of pharmacists, we propose a local standard of care led by pharmacists that incorporates patient education and the algorithm of pharmaceutical care in allergic rhinitis management. This proposed model is specifically designed for public services, including hospitals and primary care clinics. Our proposed PhaRmacISt-led Education model (AR-PRISE) aims to improve patient understanding of the overall aspects of allergic rhinitis disease and its management. Besides, this model includes patients' obtaining support from healthcare providers, particularly the pharmacist in the allergic rhinitis management. The goal of this structured care is to help patients feel more confident in their ability to deal with their long-term illness and control their symptoms as desired.

The aspect of patient education emphasises patients’ expectations of treatments, allergen identification and avoidance, and nasal product administration technique. This will be accomplished by providing patients with comprehensive written and/or audio–visual patient education.

Subsequently, patient support will be delivered via the pharmacist by following the algorithm of pharmaceutical care. This algorithm will guide the pharmacists in performing structured counselling for the patients, which includes assessing the patient’s disease severity, knowledge, and adherence to medication. Reassurance on the benefit of intranasal corticosteroids will also be provided. The pharmacist will also perform monitoring for symptom control, quality of life, and flare-up of allergic rhinitis. The pharmacist will be responsible for teaching the patient to identify the signs of allergic rhinitis flare-up and/or asthma exacerbation (if allergic rhinitis co-exists with asthma). A comparison of current practice and the proposed AR-PRISE model in overcoming these limitations is shown in Table 2 [29].

**Table 2:** Differences between current Malaysian pharmacy practices in allergic rhinitis management and the proposed AR-PRISE model

Content	Current practice of outpatient pharmacy	Proposed AR-PRISE model
Patient population	Patients with allergic rhinitis for all level of severity	Patient with moderate/severe allergic rhinitis
Human resource requirement	•Medical doctors •Pharmacists in the public services	•Medical doctors •Pharmacists in the public services
Disease identification	Diagnosis will be conducted by medical doctors	Diagnosis will be conducted by medical doctors and patient with moderate/severe allergic rhinitis is referred to the pharmacists
Symptoms control assessment	Symptoms control assessment will be performed by medical doctors	Pharmacist assess and monitor symptom control by recording: •Disease severity assessment using ARIA guidelines grading^a^ •Symptom control assessment using total nasal symptom score, and •Health-related quality-of-life assessment
Treatment	Medication will be prescribed by the medical doctors	Medication will be prescribed by the medical doctors
Patient education		
a. Disease nature	Limited/unstructured	Explaining the aetiology of allergic rhinitis and possible cure following allergy avoidance and adherence to pharmacotherapy
b. Allergen identification and avoidance	Limited/unstructured	•Explaining types of allergens •Recommending strategies in identifying and avoidance of allergens
c. Pharmacotherapy	Limited/unstructured	Explaining the indications, mode of actions and side effects of each treatment modality
d. Expectation of treatment effect	Limited/unstructured	•Explaining the optimal effect of intranasal corticosteroid can be seen in 2 weeks period •Emphasizing the importance of regular usage of intranasal corticosteroid in disease control and preventing disease progression
e. Intranasal corticosteroid usage technique	Verbal explanation in priming, administration and cleaning of nasal products (spray/drop), with the aid of product inserts/slides show when necessary	Verbal counselling in priming, administration and cleaning of nasal products (spray/drop) supplemented by written and/or audio–visual patient education material
Algorithms of pharmaceutical care in allergic rhinitis management for public services	A general counselling guideline for the pharmacist •Scope of services •General patient selection criteria •Work procedures •Assessment for medication knowledge •Technique of medication administration and medication adherence •Documentation of counselling and records management	Implementation of intensive structured counselling as specified in algorithm of pharmaceutical care (Fig. 1), on the first encounter •Patient selection criteria based on severity •Disease severity assessment and monitoring •Setting goal of treatment •Assessment for medication knowledge, treatment expectation, technique of medication administration, and medication adherence •Reassurance for the benefit of intranasal corticosteroid •Addressing concerns of intranasal corticosteroid regular usage •Teaching patient about the alert sign of asthma exacerbation and symptom flare of allergic rhinitis
Follow-up care	Counselling will be given when necessary •Mainly focusing on the technique of nasal product usage and adherence	Subsequent counselling will be performed when there is a referral by physician or when deemed necessary •Structured counselling will be applied by following the algorithm of pharmaceutical care (Fig. 1)
Patient discharge criteria	Not applicable	Aim to achieve mild allergic rhinitis based on physician judgement
General guide of pharmacotherapy in stepwise allergic rhinitis management for the knowledge of pharmacist	Limited/unstructured	Included

^a^Intermittent: < 4 days per week or < 4 weeks at a time; Persistent: ≥ 4 days per week, and ≥ 4 weeks at a time; Mild: Normal sleep, daily activities, work/school and no troublesome symptoms; Moderate to severe: One or more of the symptoms including abnormal sleep, impairment of daily activities, sport, leisure, problem at work or school, troublesome symptoms

**Fig. 1:**
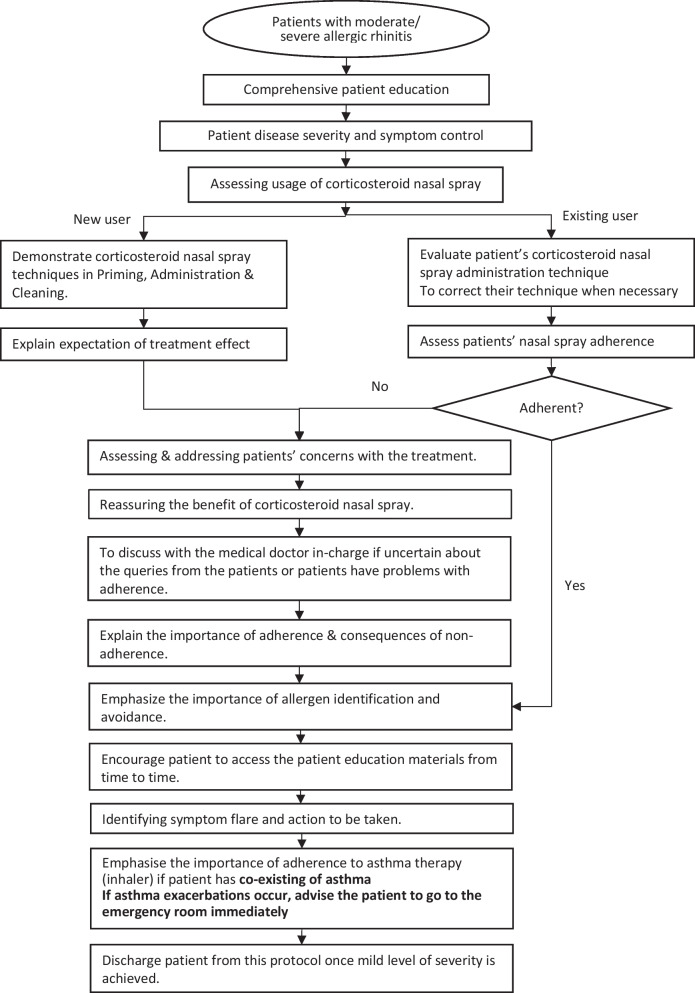
Algorithm of pharmaceutical care in the AR-PRISE model

In the AR-PRISE model, moderate/severe allergic rhinitis patients will be referred to a pharmacist by a doctor. Patients will attend a session of comprehensive patient education and structured pharmacist counselling (Fig. 1). Baseline patient information will be collected, including socio-demography, illness severity grading, current medication (prescribed and OTC allergic rhinitis treatments), and quality of life. The subsequent follow-up of patients will be conducted by the pharmacist, which can be done virtually upon referral by the physicians when deemed necessary. The patients will be counselled on specific modules of education as deemed necessary and customised according to each patient’s needs. The pharmacist will then evaluate patients' symptom control based on ARIA classifications, medication adherence, and quality of life. Should any symptoms warrant a doctor's attention, pharmacists will forward the case to the otorhinolaryngology specialist without waiting for the scheduled clinic visit.

Additional content that will enhance the understanding of the fellow pharmacists with regard to allergic rhinitis management is added to the proposed AR-PRISE model. The contents include: the introduction of the disease; the burden to the health system caused by the disease; objectives of the protocol; scope of services; human resources requirements; procedures of execution (patient selection, clinic operation, and pharmacist's responsibilities); non-pharmacological management; duration of visit; patient discharge criteria; documentation; and patient outcome measures. A stepwise management of allergic rhinitis treatment will be added to give the pharmacist a clearer picture of how to manage this disease.

## Implementation of the AR-PRISE model

Endorsing the AR-PRISE model into practise requires collaboration between the pharmacy services and otorhinolaryngology services in the Ministry of Health. This involves holding a large-scale workshop to introduce this model to pharmacists. Evidence-based guidance for pharmacists should be provided with the goal of transferring the latest research findings into pharmacy practices.

## Conclusions

The burden of morbidity and disability caused by allergic rhinitis has been underappreciated by healthcare providers and patients. Pharmacists are well-positioned to play an important role in the management of allergic rhinitis in Malaysian public healthcare institutions. The proposed AR-PRISE model portrays pharmacists' duties in allergic rhinitis management and would expand their public service role. Comprehensive patient education in the model would help the pharmacists to provide structured and standardised information. There is an urgent need for a multi-stakeholder approach to successfully implement this pharmacist-led educational model, which could result in better patient outcomes and burden reduction from allergic rhinitis management.

## Data Availability

No data collection is involved in the write-up of this article.
